# Self-reported weight loss practices in professional mixed martial artists – preliminary findings

**DOI:** 10.1186/1550-2783-10-S1-P14

**Published:** 2013-12-06

**Authors:** Paul La Bounty, Elfego Galvan, Jeremy Reid, Bill I Campbell, Jeremy McElroy, Eva Doyle, Tony Boucher

**Affiliations:** 1 Baylor University – Waco, TX, USA; 2 Texas A&M University – College Station, TX, USA; 3 University of South Florida – Exercise and Performance Nutrition Laboratory, Tampa, FL, USA

## Background

Mixed Martial Arts (MMA) is a physiologically demanding sport that requires athletes to compete in weight restricted classes. As a result, it is a common practice for many athletes competing in this sport to undergo weight loss prior to competition. These practices included various dieting strategies to lose weight over a period of days to weeks as well as mild to severe losses of body water in close proximity to the official “weigh ins.” The purpose of this ongoing study is to examine self-reported weight loss strategies among professional mixed martial artists.

## Methods

Male professional mixed martial artists between the ages of 18-50 years old were eligible to participate in this ongoing study. The participants were recruited and interviewed at various locations in the states of Texas and Nevada using a newly developed questionnaire. The questionnaire was initially reviewed for content by three exercise physiologists and two registered dietitians with significant knowledge of sports nutrition. During the interview, the questions were read out loud to the participants. The participants were also given a copy of the questionnaire so they could read along as the questions were being asked. If the self-reported response was give as a range, the averages between the two values were utilized. Averages and standard deviations were calculated using Microsoft Excel.

## Results

All data are presented in means and standard deviations. To date, 16 athletes (age = 29.9 ± 5.1 years old; years fighting professionally= 5.9 ± 5.1) have completed in the study. Of the 16 participants, only 5 of 8 possible weight classes are represented [featherweights (FW) = 145 lbs; lightweights (LW) = 155lbs; welterweights (WW) = 170 lbs; light heavyweights (LHW) = 205 lbs; and heavyweights (HW) < 260 lbs]. Only one heavyweight completed the study and as a result, no SD is included for those values. On average, FW, LW, WW, LHW, and HW, reported losing ~ 27.5 ± 17.7, 22.6 ±5.4, 24.2 ± 9.8, 17.6 ± 2.8, and 10 lbs, respectively, during their typical training camps leading up to a fight. When asked what was the maximum amount of weight that was reduced in the 48 hours prior to the official “weigh ins”, FW, LW, WW, LHW, and HW, reported losing a maximum of ~ 11.5 ± 9.2, 14 ± 2.2, 14.2 ± 5.8, 16.3 ± 7.6, and 0.0 lbs, respectively. Lastly, all participants in every weight class, reported using either Pedialyte ® or Gatorade ®, either exclusively or in conjunction with another fluid (i.e., water, apple juice, etc.) to rehydrate immediately following the official weigh-ins.

## Conclusion

The results of this ongoing study indicate that during “training camps” leading up to a scheduled fight, professional mixed martial artists often lose significant amounts of body weight. Furthermore, the common practice of cutting water weight in the days leading up to the weigh-ins can be significant and potentially dangerous. As a result, more research is needed to elucidate safe and effective ways to lose weight in professional mixed martial artist prior to competition.

